# Alpha-Beta Hybrid Quantum Associative Memory Using Hamming Distance

**DOI:** 10.3390/e24060789

**Published:** 2022-06-04

**Authors:** Angeles Alejandra Sánchez-Manilla, Itzamá López-Yáñez, Guo-Hua Sun

**Affiliations:** 1 Centro de Investigación en Computación, Instituto Politécnico Nacional, Unidad Profesional Adolfo López Mateos, Juan de Dios Bátiz s/n esq. Miguel Othón de Mendizábal, Mexico City 07700, Mexico; 2 Centro de Innovación y Desarrollo Tecnológico en Cómputo, Instituto Politécnico Nacional, Unidad Profesional Adolfo López Mateos, Juan de Dios Bátiz s/n esq. Miguel Othón de Mendizábal, Mexico City 07700, Mexico

**Keywords:** quantum associative memory, hamming distance, quantum machine learning, pattern recognition, Alpha-Beta associative model

## Abstract

This work presents a quantum associative memory (Alpha-Beta HQAM) that uses the Hamming distance for pattern recovery. The proposal combines the Alpha-Beta associative memory, which reduces the dimensionality of patterns, with a quantum subroutine to calculate the Hamming distance in the recovery phase. Furthermore, patterns are initially stored in the memory as a quantum superposition in order to take advantage of its properties. Experiments testing the memory’s viability and performance were implemented using IBM’s Qiskit library.

## 1. Introduction

Quantum computing is an emerging area of computing based on the principles of quantum mechanics. In it, information is represented by quantum states, generally using qubits as the basic unit of storage, and the properties of quantum mechanics such as superposition, are exploited in order to improve the processing speed. Consequently, in recent years, there has been an increasing interest for new developments in the field [1,2,3]. One of these new developments is the creation of quantum algorithms that can provide a quadratic or even an exponential speed-up compared to their classical counterparts. Among the best known quantum algorithms are those of Shor and Grover et al. [4,5], of which different versions have been developed for several areas. Quantum machine learning is one of the particular interests for this work.

Machine learning (ML) is based on the minimization of a constrained multivariate function, and its algorithms are used for data mining and data visualization techniques [6]. In particular, associative memories are a specific class of machine learning algorithms that perform the task known as “retrieval”. In order to take advantage of quantum computing, the idea of a quantum associative memory (QAM) arose similarly to a Hopfield network, although its quantum formulation did not require a reference to artificial neurons [7].

To date, there exist some proposals of quantum associative memories; however, it is worth commenting that the first time they were mentioned was by Ventura et al. in [7], where Grover’s algorithm is used as the main approach. Subsequently, other approaches have been used: quantum associative memories including linear and nonlinear algorithms by Zhou et al. [8], in which the quantum matrix is constructed using binary decision diagrams; in [9], the authors proposed a QAM that performs image recognition for face detection using the Gabor transform; multidirectional associative memories [10,11,12], where fuzzy inference is used, and by means of different layers, it is able to be noise tolerant; in another interesting work [13], the authors propose a QAM that is used as a tool for medical personnel to obtain the diagnosis of four tropical diseases.

In this work, a hybrid quantum associative memory (HQAM) is proposed, using the Alpha-Beta support vector machine [14,15] at the learning phase and the quantum Hamming distance subroutine at the retrieval phase. It should be mentioned that it is considered hybrid because it combines both classical and quantum computing to obtain the greatest potential of both parts.

The advantage of this model over a classical model lies in the reduction of operations at the recovery or retrieval phase when calculating the Hamming distance, since it takes advantage of the parallelism that allows the superposition of patterns in the trained memory.

## 2. Materials and Methods

### 2.1. Basic Concepts on Associative Memories

Some of the basic concepts regarding the theory of associative memories in classical computing are explained below. Their operation is divided into two phases:

1. Learning phase (generation)
2. Retrieval phase (operation)

The fundamental purpose of an associative memory is to correctly recall complete patterns from possibly altered input patterns as this is the most attractive feature of associative memories. In the particular case where the patterns have only binary data, the only possible types of alterations (i.e., noise) are additive, subtractive or mixed. Thus, an associative memory **M** can be formulated as an input-output system [16]:

(1) $$x\to \begin{array}{c} M \end{array}\to y$$

The input pattern is represented by a column vector, denoted by *x*, and the output pattern by a column vector, denoted by *y*. Each of the input patterns form an association with the corresponding output pattern, the notation for this association is similar to that of an ordered pair, e.g., the *x* and *y* patterns in the schematic form the association (*x*, *y*); a specific association is denoted as (*x^k^*, *y^k^*), where *k* is a positive integer. The associative memory **M** is represented by a matrix whose *i* *j* component is denoted as *m_ij_* [17]. The matrix **M** is generated from a finite set of associations that is known as the fundamental set of associations or simply as the fundamental set. The cardinality of the fundamental set is denoted by *p*. If *μ* is an index, the fundamental set is represented as follows:

(2) $$\left\{\left(x^{\mu },y^{\mu }\right)|\mu \;=\;1,2,\ldots ,p\right\}$$

The patterns that form the associations of the fundamental set are called fundamental patterns. If it holds that *x^μ^* = *y^μ^*, for ∀*μ* ∈ {1, 2, …, *p*}, the memory is said to be autoassociative, otherwise, it is called heteroassociative. It is possible for the fundamental patterns to be altered by different types of noise, thus, in order to differentiate an altered pattern from its fundamental counterpart, a tilde is used over the pattern, that is, ${\tilde{x}}^{k}$ denotes an altered version of the fundamental pattern *x^k^*. When the memory **M** receives an altered pattern ${\tilde{x}}^{\omega }$ as input (*ω* ∈ {1, 2, …, *p*}, and **M** responds with the corresponding fundamental output pattern *y^ω^*, it is said that the memory recall is correct.

Two sets **A** and **B**, need to be specified; the components of the column vectors, representing both input and output patterns, are elements of the set **A**, whereas the components of the matrix **M** are elements of **B**. There are no prerequisites or limitations on the choice of these two sets, so they do not necessarily have to be different or have special characteristics.

The positive integer numbers *n* and *m* are used to represent the dimension of the input and output patterns respectively. Then the fundamental input and output pattern can be represented as:

(3) $$x^{\mu }\;=\;{\left(x_{1}^{\mu },x_{2}^{\mu },\ldots ,x_{n}^{\mu }\right)}^{t}\;=\;\left(\begin{array}{c} x_{1}^{\mu }\\ x_{2}^{\mu }\\ \vdots \\ x_{n}^{\mu } \end{array}\right)\in \;A^{n}$$

(4) $$y^{\mu }\;=\;{\left(y_{1}^{\mu },y_{2}^{\mu },\ldots ,y_{m}^{\mu }\right)}^{t}\;=\;\left(\begin{array}{c} y_{1}^{\mu }\\ y_{2}^{\mu }\\ \vdots \\ y_{m}^{\mu } \end{array}\right)\in \;A^{m}$$

### 2.2. Alpha-Beta HQAM Algorithm

**Figure 1:**
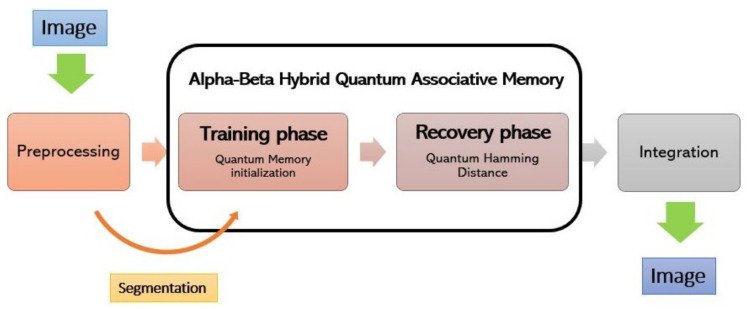
Alpha-Beta HQAM model architecture.

The architecture of the proposed Alpha-Beta HQAM model is shown in Figure 1, and the detailed algorithm flow is summarized as following the figure:

1. Preprocessing is performed to reduce the number of qubits required. Based on the Alpha-Beta SVM associative model, it is intended to take advantage of only that information which is not repeated in the patterns of the fundamental set. At result of this point, there will be two fundamental sets: the restricted fundamental set and the negated restricted fundamental set, as is further explained bellow.
2. A segmentation of the patterns with a length of *n =* 4 is performed, assuming that they are binary images (0 white, 1 black). Once this is completed, partial fundamental sets are formed to start training the Alpha-Beta HQAM. Its principal objective is to mitigate the limited number of available qubits and to make it feasible to run experiments using Qiskit SDK.
3. The training set is encoded into a superimposed quantum state with the equal probability amplitudes: $|m\rangle \;=\;\frac{1}{\sqrt{n}}\sum_{i\;=\;1}^{n}|x^{i}\rangle$, where *x_i_* is the *i*-th binary pattern of length *n*. The storage complexity of this algorithm is a linear function related to the number of patterns in the training set.
4. The retrieval algorithm computes the Hamming distance between the input and all overlapping patterns in the quantum state of the memory. It indicates the probability that an input pattern is in memory, based on the results of its distance distribution over all stored patterns at once. This algorithm is described in Section 2.4. This process must be repeated several times until all the subsets of the training set have been recovered.
5. Once the previous step is finished, the retrieval for each segment (obtained in step 2) of the patterns in the training set is performed and reintegrated to get the original length of the patterns.

### 2.3. Preprocessing Module

In this first part, the information is preprocessed to reduce its volume and keep only the most relevant information in the fundamental patterns, this is based on the Alpha-Beta SVM associative model [18], the details are as follows.

#### 2.3.1. The *α* and *β* Operators

In this model, the main mathematics used are binary operators designed for the original Alpha-Beta associative memories [15]. They are based on two binary operators: the *α* operator used in the learning phase and the *β* operator for the retrieval phase.

The sets are defined **A** = {0, 1} and **B** = {0, 1, 2}, then: the binary operator *α*: **A** × **A** → **B** and the binary operator *β*: **B** × **A** → **A** are defined in Table 1, where ∨ is the *max* operator and ∧ is the *min* operator. The sets **A** and **B**, the operators *α* and *β*, together with the operators ∧ and ∨, form the algebraic system that is the mathematics basis for Alpha-Beta Associative Models.

#### 2.3.2. Alpha-Beta SVM Associative Model

Some important definitions within the Alpha-Beta SVM associative model are explained below.

A zero vector is defined as the vector whose components are all of value 0, and is denoted **0**. A one vector is defined as the vector whose components are all of value 1, and is denoted by 1.

Let **A** = {0, 1} be a binary set, *x* and *y* are two vectors, *x,y* ∈ *A^n^*, n ∈ $Z^{+}$, with *x,y* ≠ **0**,**1**. The auxiliary *c* and *k_i_* are defined as:

(5) $$c\;=\;\wedge_{j\;=\;1}^{n}\;j,\;\mathrm{where}\;x_{j}\;=\;1\;\mathrm{and}k_{i}\;=\;\sum_{1}^{i}x_{i},\;\mathrm{where}\;i\;\in \;\left\{1,2,\ldots ,n\right\}.$$

with *k* being a positive integer number which can have a value between 1 and *r* inclusive.

- Elimination: The elimination of the vector *y* with respect to the vector *x* can be obtained as follows:
  1. Define the index *c* according to Equation (5);
  2. Eliminate the components *x_c_* and *y_c_*;
  3. Decrease the indices of the *x_i_* and *y_i_*, where *c* < *i* ≤ *n* if they exist;
  4. Obtain the eliminated vectors *ε*_1_(*x*) and *ε*_1_(*y*), the dimension of the vectors are decreased to *n* − 1.
  If the elimination in *y* according to *x* is applied continually with:
  (6) $$c\;=\;\wedge_{j\;=\;1}^{n-i}j\;\mathrm{where}\;{\left[\varepsilon_{i}\left(x\right)\right]}_{j}\;=\;1,0\;\leq \;i\;\leq \;k_{n}$$
  in the *k*-th iteration, the transformed vectors are denoted *ε_k_*(*x*) and *ε_k_*(*y*).
- Restriction: Continue to repeat the elimination, until the valid *k*-th iteration, in other words, *ε*_(*k *− 1_(*x*) ≠ 0, and*ε_k_*(*x*) = 0, we get a new vector $y\;\in \;A^{n\;-\;k_{n}}$, which is the restriction of *y* with respect to *x*, denoted by ${y|}_{x}$. In this definition, the vector *y* is really eliminated by the corresponding component with respect to those components of the vector *x* with a value of 1
  Let $x\;=\;\left(\begin{array}{c} 1\\ 0\\ 1\\ 0 \end{array}\right)$ and $y\;=\;\left(\begin{array}{c} 0\\ 1\\ 1\\ 1 \end{array}\right)$; obtain ${y|}_{x}$:
  (7) $$\overset{c\;=\;1}{\to }\varepsilon_{1}\left(x\right)\;=\;\left(\begin{array}{c} 0\\ 1\\ 0 \end{array}\right),\varepsilon_{1}\left(y\right)\;=\;\left(\begin{array}{c} 1\\ 1\\ 1 \end{array}\right)\overset{c\;=\;3}{\to }\varepsilon_{2}\left(x\right)\;=\;\left(\begin{array}{c} 0\\ 0 \end{array}\right),\varepsilon_{2}\left(y\right)\;=\;\left(\begin{array}{c} 1\\ 1 \end{array}\right)$$
  Therefore ${y|}_{x}\;=\;\varepsilon_{2}\left(y\right)\;=\;\left(\begin{array}{c} 1\\ 1 \end{array}\right)$.

Let **A** = {0, 1} be a binary set, *x* and *z* are two vectors *x* ∈ *A_n_*, *z* ∈ *A_m_*, *n*, $m\;\in \;Z^{+}$ and *m* = *n* − *k_n_*, with *x,z *≠ 0, 1..

- Insertion: The insertion of the vector *z* with respect to the vector *x* can be obtained as follows:
  1. Define the index *c* according to Equation (5);
  2. Shift the components *z_i_* to *z*_*i*+1_, where *c* ≤ *i* ≤ *m*;
  3. Insert *z_c_* = 1, assign *x_c_* = 0;
  4. Obtain the inserted vectors *I*_1_(*x*) and *I*_1_(*y*), the dimension of the vector *z* increased to *m* + 1.
  If the insertion in *z* according to *x* is applied continually with:
  (8) $$c\;=\;\wedge_{j\;=\;1}^{n-i}j\;\mathrm{where}\;{\left[I_{i}\left(x\right)\right]}_{j}\;=\;1,0\leq i\leq k_{n}$$
  in the *k*-th iteration, the transformed vectors are denoted *I_k_(x)* and *I_k_(z)*.
- Expansion: Continue to repeat the insertion, until the valid *k*-th iteration, in other words, *I*_(*k* − 1)_(*x*) ≠ 0, and *I_k_(x)* = 0, we get a new vector *z* ∈*A^n^*, which is the expansion of *z* with respect to *x*, denoted by ${z|}^{x}$. In this definition, the vector *z* is really inserted with a value of 1 to the corresponding position with respect to those components of the vector *x* with a value of 1. For example,
  Let $x\;=\;\left(\begin{array}{c} 1\\ 1\\ 0\\ 0 \end{array}\right)$ and $y\;=\;\left(\begin{array}{c} 1\\ 0 \end{array}\right)$; obtain ${y|}^{x}$:
  (9) $$\overset{c\;=\;1}{\to }I_{1}\left(x\right)\;=\;\left(\begin{array}{c} 0\\ 1\\ 0\\ 0 \end{array}\right),I_{1}\left(y\right)\;=\;\left(\begin{array}{c} 1\\ 1\\ 0 \end{array}\right)\overset{c\;=\;2}{\to }I_{2}\left(x\right)\;=\;\left(\begin{array}{c} 0\\ 0\\ 0\\ 0 \end{array}\right),I_{2}\left(y\right)\;=\;\left(\begin{array}{c} 1\\ 1\\ 1\\ 0 \end{array}\right)$$
  Therefore ${y|}^{x}\;=\;I_{2}\left(y\right)\;=\;\left(\begin{array}{c} 1\\ 1\\ 1\\ 0 \end{array}\right)$.
- Support Vector: Let **A** = {0, 1}, be the binary set, let *x* be a vector *x* ∈ *A_n_* of dimension $n\in Z^{+}$, and $p\in Z^{+}$, $1<p\leq 2^{n}$ is the cardinality of the fundamental set of an Alpha-Beta SVM associative model. The vector *S* is made up of *n* binary components, which are calculated from the fundamental set as:
  (10) $$S_{i}\;=\;\left\{\begin{array}{cc} \wedge_{k\;=\;1}^{p/2}\beta \left(x_{i}^{2k-1},x_{i}^{2k}\right) & \mathrm{if}\;p\;\mathrm{is}\;\mathrm{even},\\ \beta \left[\wedge_{k\;=\;1}^{\frac{\left(p-1\right)}{2}}\beta \left(x_{i}^{2k-1},x_{i}^{2k}\right),x_{i}^{p}\right] & \mathrm{if}\;p\;\mathrm{is}\;\mathrm{odd} \end{array}\right.$$

Returning to the current proposal, based on the fundamental set and using Equation (10), a pattern can be obtained that contains the repeated information in all the fundamental patterns, subsequently, it is eliminated from the fundamental set, leaving only the information that differentiates a fundamental pattern from all the others. This repeated information is stored in a vector called Support Vector (*S*).

Since we are looking for the information that can be more significant in the patterns, the next step is to negate the fundamental set and repeat the procedure described in the previous paragraph, thus obtaining the Negated Support Vector $\left(\hat{S}\right)$.

**Figure 2:**
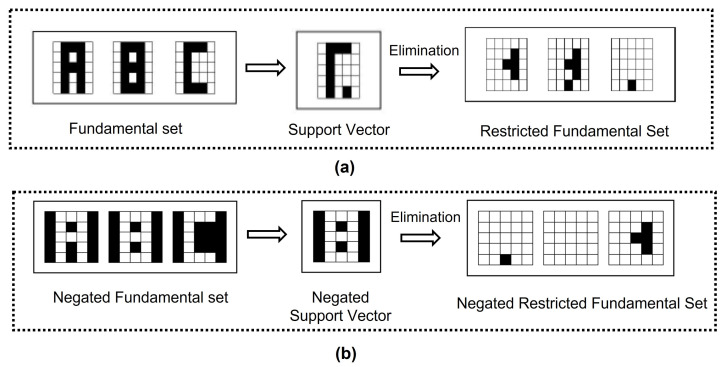
Alpha-Beta HQAM preprocessing: (**a**) Restricted Fundamental Set. (**b**) Negated Restricted Fundamental Set.

By the end of this part, there are two fundamental sets: the restricted fundamental set and the negated restricted fundamental set. Figure 2 shows an example of pattern reduction on a fundamental set and Algorithm 1 shows the process of obtaining the fundamental sets:
**Algorithm 1** Alpha-Beta HQAM preprocessing1: From the Fundamental set, calculate Support Vector (*S*) as shown in Equation (10).2: For each $\mu \in \left\{1,2,\ldots ,n\right\}$, obtain $x^{\mu }{|}_{S}$. From these results Restricted Fundamental Set is obtained.3: For each $\mu \in \left\{1,2,\cdots ,n\right\}$, obtain $\bar{x^{\mu }}$ who is the Negated Fundamental Set.4: From Negated Fundamental set, calculate Negated Support Vector $\left(\hat{S}\right)$. Equation (10)5: For each $\mu \in \left\{1,2,\cdots ,n\right\}$, obtain $\bar{x^{\mu }}{|}_{\hat{S}}$. From these results Negated Restricted Fundamental Set is obtained.


### 2.4. Segmentation

At present, commercial quantum computers do not yet exist, however, some are available through platforms such as the IBM Q Experience, where up to five qubits can be freely used. Therefore, in order to be able to recover images that exceed this capacity, in the proposed Alpha-Beta HQAM model the images are segmented into 4-bit patterns considering that they are binary images (0 white and 1 black) to reduce their length.

**Figure 3:**
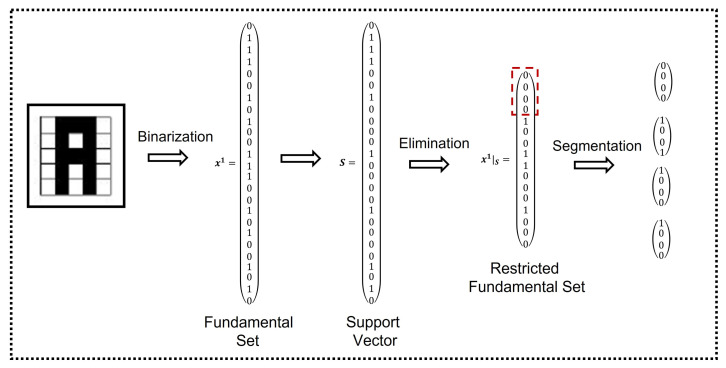
Alpha-Beta HQAM segmentation.

Figure 3 shows an example of the segmentation process of the Restricted Fundamental Set length, which was calculated as explained in Section 2.3.2, and repeated for the Negated Restricted Fundamental Set.

Once this segmentation is completed, fundamental partial sets are formed to start training the proposed model.

### 2.5. Training Phase

To use some of the properties of quantum mechanics in this model, it is necessary to pass the fundamental ensemble to the quantum state. That is, to transform the bits into qubits to take advantage of the superposition property. The first step is to obtain the unique patterns of the fundamental sets so that the probabilities are distributed and thus there is no class imbalance. This is undertaken to avoid that the probability is biased and, when retrieving it, the majority class is always taken as the result. At this phase, memory initialization is carried out, which consists of an operation that transforms the training set into quantum states with the same probability. Given *p* binary patterns $x^{i}$ of length *n*, the memory represented as a quantum state is shown as:

(11) $$|m\rangle \;=\;\frac{1}{\sqrt{n}}\sum_{i\;=\;1}^{n}|x^{i}\rangle$$

The complexity of this algorithm is linear as a function of the number of patterns in the training set.

A detailed description of how the storage is performed with an *n* bits pattern dataset as input is given below. Algorithm 2 shows the necessary gates to perform each of the steps:

1. Three registers are used: first register *x* of *n* qubits in which the patterns $x^{i}$ to store are represented.
2. An ancilla register *u* of two qubits sets up in |01〉 state.
3. Another register *m* of *n* qubits to store the memory, the order is from right to left which is initially prepared in $|0_{1},\ldots ,0_{n}\rangle$ state.
4. The second qubit of the register *u*, $u_{2}$, in state, |0〉 for the stored patterns and the first qubit in |1〉 for the processing term.
5. For every pattern $x^{i}$ in the training set to be stored, if the contents of the patterns and the memory registers are identical, all these qubits will be transformed in |1〉’s.
6. The first ancilla qubit $u_{1}$ of the processing term is transformed from |0〉, leaving it unchanged for the stored patterns term.
7. The input pattern $x^{i}$ is added to the memory register with uniform amplitudes. This is carried out by applying the $CS^{i}$ gate, as shown below:
  (12) $$\begin{array}{c} CS^{i}\;=\;|0\rangle \langle 0|⊗I+|1\rangle \langle 1|⊗S^{i}\\ \mathrm{where}\;S^{i}\;=\;\left(\begin{array}{cc} \sqrt{\frac{i-1}{i}} & \frac{1}{\sqrt{i}}\\ \frac{-1}{\sqrt{i}} & \sqrt{\frac{i-1}{i}} \end{array}\right) \end{array}$$

Further steps apply inverse operations to return the memory to its initial state and prepare it to receive the next pattern. This algorithm runs several times until all the patterns have been processed and stored on |M〉.
**Algorithm 2** Storage algorithm [19]1: Prepare the initial state $|\psi_{0}^{i}\rangle \;=\;|0_{1},\ldots ,0_{n};01;0_{1},\ldots ,0_{n}\rangle$2: **for each **
$x^{i}\in$ datum **do**3:   Load $x^{i}$ into quantum register ${|x\rangle }_{n}$4:   $|\psi_{1}^{i}\rangle \;=\;\prod_{j\;=\;1}^{n}2CNOT_{x_{j}^{i}u_{2}m_{j}}|\psi_{0}^{i}\rangle$5:   $|\psi_{2}^{i}\rangle \;=\;\prod_{j\;=\;1}^{n}Xm_{j}CNOT_{x_{j}^{i}m_{j}}|\psi_{1}^{i}\rangle$6:   $|\psi_{3}^{i}\rangle \;=\;nCNOT_{m_{1},...,m_{n}u_{1}}|\psi_{2}^{i}\rangle$7:   $|\psi_{4}^{i}\rangle \;=\;CS_{u_{1}u_{2}}^{x+1-i}|\psi_{3}^{i}\rangle$8:   $|\psi_{5}^{i}\rangle \;=\;nCNOT_{m_{1},...,m_{n}u_{1}}|\psi_{4}^{i}\rangle$9:   $|\psi_{6}^{i}\rangle \;=\;\prod_{j\;=\;n}^{1}CNOT_{x_{j}^{i}m_{j}Xm_{j}}|\psi_{5}^{i}\rangle$10:    $|\psi_{7}^{i}\rangle \;=\;\prod_{j\;=\;n}^{n}2CNOT_{x_{j}^{i}u_{2}m_{j}}|\psi_{6}^{i}\rangle$11:    Unload $x^{i}$ from quantum register ${|x\rangle }_{n}$12: **end for**


### 2.6. Retrieval Phase

The Hamming distance [20] in classical computing is defined as the count of the number of bits that are different between two patterns of equal length, for example, 0110↔0001 has a distance of three. However there is a quantum version of this algorithm that was proposed by Trugenberger [21] where the output is determined by a probability distribution in the memory that has a peak around the stored patterns that are closest with respect to the Hamming distance of the input. If the input pattern is far away from the patterns stored in memory, |1〉 will be obtained as output. Otherwise, |0〉 would be obtained.

The following is a detailed description of how retrieval is carried out. Algorithm 3 shows the necessary gates to perform each of the steps.

1. In the initial step of the algorithm, an overlay of the training set containing the training data is constructed:
  (13) $$|M\rangle \;=\;\frac{1}{\sqrt{N}}\sum_{p}^{}|x_{1}^{p}\ldots x_{n}^{p},c^{p}\rangle ;$$
2. Starting from this, construct the initial state:
  (14) $$|\psi_{0}\rangle \;=\;\frac{1}{\sqrt{N}}\sum_{p}^{}|\tilde{x_{1}},\ldots ,\tilde{x_{n}};x_{1}^{p},\ldots ,x_{n}^{p};c^{p}\rangle ;$$
3. The initial state consists of three registers, the first contains the input pattern, the second contains the memory |M〉, and the third contains the ancilla qubit set to zero. During the first step, the ancilla is a superposition through the Hadamard gate, giving rise to:
  (15) $$|\psi_{1}\rangle \;=\;\frac{1}{\sqrt{N}}\sum_{p}^{N}|\tilde{x_{1}},\ldots ,\tilde{x_{n}};x_{1}^{p},\ldots ,x_{n}^{p};c^{p}\rangle ⊗\frac{1}{\sqrt{2}}\left(|0\rangle +|1\rangle \right);$$
4. Next, by applying a CNOT gate to all *j* components of the patterns (${\tilde{x}}_{j}$ is the control qubit and $x_{j}$ is the target qubit), followed by an X gate to each of the target qubits, the following is obtained
  (16) $$|\psi_{2}\rangle \;=\;\frac{1}{\sqrt{N}}\sum_{p}^{N}|\tilde{x_{1}},\ldots ,\tilde{x_{n}};d_{1}^{p},\ldots ,d_{n}^{p};c^{p}\rangle ⊗\frac{1}{\sqrt{2}}\left(|0\rangle +|1\rangle \right);$$
5. Applying unitary operator $U\;=\;exp\left(i\frac{\pi }{2n}\hat{H}\right)$, where H is a hamiltonian summing over all the components $d_{j}$, calculate the Hamming distance between $\tilde{x}$ and $x_{j}$, which obtains:
  (17) $$\begin{array}{c} |\psi_{3}\rangle \;=\;\frac{1}{\sqrt{2N}}\sum_{p}^{N}exp\left[i\frac{\pi }{2n}d_{h}\left(\tilde{x},x^{p}\right)\right]|\tilde{x_{1}},\ldots ,\tilde{x_{n}};d_{1}^{k},\ldots ,d_{n}^{p};c^{p};0\rangle \;\\ +\frac{1}{\sqrt{2N}}\sum_{p}^{N}exp\left[-i\frac{\pi }{2n}d_{h}\left(\tilde{x},x^{p}\right)\right]|\tilde{x_{1}},\ldots ,\tilde{x_{n}};d_{1}^{k},\ldots ,d_{n}^{p};c^{p};1\rangle ; \end{array}$$
6. Applying Hadamard gate on the last qubit, is obtaining the next state:
  (18) $$\begin{array}{c} |\psi_{4}\rangle \;=\;\frac{1}{\sqrt{N}}\sum_{p}^{N}cos\left(\frac{\pi }{2n}d_{h}\left(\tilde{x},x^{p}\right)\right)|\tilde{x_{1}},\ldots ,\tilde{x_{n}};d_{1}^{p},\ldots ,d_{n}^{p};c^{p};0\rangle \\ +\frac{1}{\sqrt{N}}\sum_{p}^{N}sin\left(\frac{\pi }{2n}d_{h}\left(\tilde{x},x^{p}\right)\right)|\tilde{x_{1}},\ldots ,\tilde{x_{n}};d_{1}^{p},\ldots ,d_{n}^{p};c^{p};1\rangle ; \end{array}$$
7. Qubits |x〉 and |c〉 need to be measured.
  By measuring |x〉 and |c〉 it is possible to know which pattern is the result of the retrieval, unlike the original version of the algorithm, we only can know whether the pattern to be retrieved belongs to the trained memory or not.

**Algorithm 3:** Quantum Hamming Algorithm1: Load the input *p* pattern in the quantum register |i〉2: $|\psi_{0}\rangle \;=\;\frac{1}{\sqrt{N}}\sum_{p}^{}|\tilde{x_{1}},\ldots ,\tilde{x_{n}};x_{1}^{p},\ldots ,x_{n}^{p};c^{p}\rangle$3: $|\psi 1\rangle \;=\;H_{c}\prod_{j\;=\;n}^{1}|\psi_{0}\rangle$4: $|\psi_{2}\rangle \;=\;\prod_{j\;=\;1}^{n}X_{m_{j}}CNOT_{i_{j},m_{j}}|\psi_{0}\rangle$5: $|\psi_{3}\rangle \;=\;\prod_{i\;=\;1}^{n}{\left(CU^{-2}\right)}_{c,m_{i}}\prod_{j\;=\;1}^{n}U_{m_{j}}|\psi_{1}\rangle$6: $|\psi 4\rangle \;=\;H_{c}\prod_{j\;=\;n}^{1}CNOT_{i_{j},m_{j}}X_{m_{j}}|\psi_{2}\rangle$7: Measure qubits |x〉 and |c〉8: **if** c == 0 **then**9:   Measure the memory to obtain the desired state.10:  **end if**

### 2.7. Validation Methods

To evaluate the capacity of retrieval of the model proposed in this work, two validation methods belonging to the state of the art of machine learning were used, which are explained below.

- Resubstitution Error (RE): In this method the test set is the same as the training set [22], the formula for calculating it is:
  (19) $$RE\;=\;\frac{\mathrm{errors}}{\mathrm{number}\;\mathrm{of}\;\mathrm{patients}}$$
- Leave One Out: Is a special case of cross-validation where the number of folds equals the number of instances in the dataset. Thus, the learning algorithm is applied once for each instance, using all other instances as a training set and using the selected instance as a single-item test set [23].

## 3. Results and Discussion

**Figure 4:**
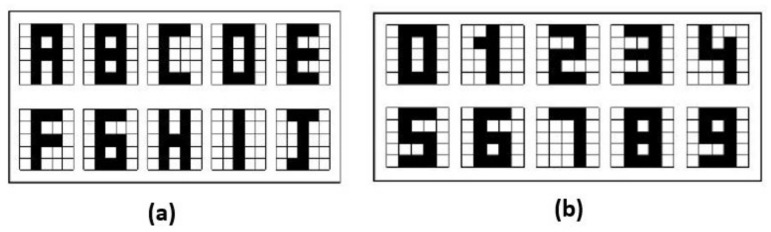
Original datasets: (**a**) Letters dataset. (**b**) Digit numbers dataset.

In this section the results obtained in the proposed Alpha-Beta HQAM model are presented and analyzed. Two datasets were chosen, the first one is a set of 10 letters of the alphabet as shown in Figure 4, each of the letters consists of a 5 × 5 pixels image that is binarized to apply the model, considering one to the black pixel and zero to the white pixel. The second one is the digit numbers shown in Figure 4.

For the execution of the experiments, Python language is used with the Qiskit SDK to simulate quantum circuits in a computer. Remembering that the proposal is hybrid, the preprocessing and segmentation part is performed with Python programming and the associative memory phases with the Qiskit SDK. Appendix A shows the code that was used to run the experiments presented below.

The experimental results with these two datasets are presented in five parts. First, the preprocessing of each of the datasets is described. Then, in the second subsection, a summary of the results obtained with the resubstitution error validation method is shown. In the third subsection, the results with the Leave One Out validation method are reported, as well as their corresponding discussion. The fourth subsection shows the result of applying the three types of noise (Additive, Subtractive and Mixed) to the first database and finally in the last subsection a summary of the results and their corresponding discussions are represented.

### 3.1. Dataset Preprocessing

When preparing the fundamental set, it is composed of 10 patterns and each one has 25 features, then the Support Vector and the Negated Support Vector are calculated and as a result the restricted fundamental sets are obtained.

For the experiment using resubstitution error as the validation method in both datasets, the number of features has no variation on its original state In Table 2 and Table 3 the respective results are shown.

On the other hand, for the experiment using Leave One Out as the validation method, the number of features varies between each letter or number depending on the datasets, the respective results are shown in Table 4 and Table 5.

At the end of this part, the segmented training sets are formed into 4 bits that upon application of the initialization algorithm become qubits.

### 3.2. Results with the Resubstitution Error as Validation Method

**Figure 5:**
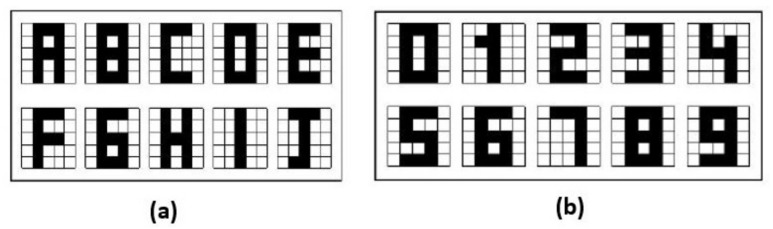
Retrieval results with Resubstitution Error: (**a**) Letters dataset. (**b**) Digit numbers dataset.

For this validation method, the retrieval is accurate for each of the images of both fundamental sets in the proposed model. In Figure 5 the results are shown for both datasets, with this it can be said that its forgetting factor is zero. That is, the patterns with which the quantum associative memory is already trained are no longer forgotten during the retrieval phase.

### 3.3. Results with Leave One out as Validation Method

**Figure 6:**
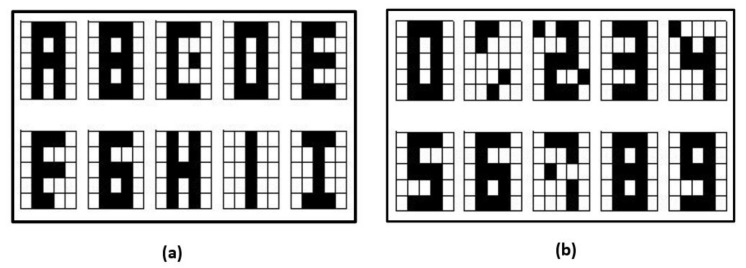
Retrieval results with Leave One Out: (**a**) Letters dataset. (**b**) Digit numbers dataset.

For this experiment, the results obtained are shown in Figure 6. For these cases, the retrieval could be obtained for most of the images, for the letters there were seven of a set of ten and for the digits six of ten. Notice that although it was not complete in the other cases they were very close approximations.

In the case of the letter dataset, since most of the letters resemble each other, it generates a little confusion in some pixels, perhaps this depends on the length that was chosen for the patterns and does not allow one to have considerably different training from each other.

Analyzing the results of the dataset of numbers, the one with the highest error is one, this may be happening because the other digits are very different from it, so in the learning phase it does not have enough training to obtain a better retrieval. These latter two hypotheses merit further analysis.

**Figure 7:**
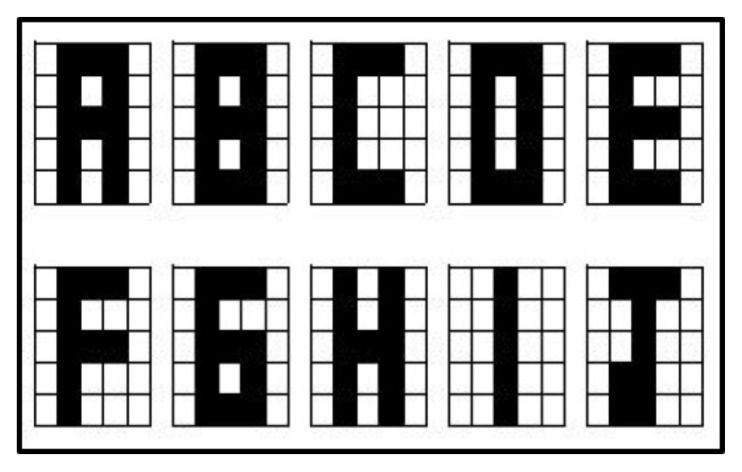
Dataset of letters with different J.

In addition, the results were compared with those obtained in Neto et al. [24], they designed a quantum associative memory, where the retrieval phase consists of three parts: Exchange, Quantum Fourier Transform and Grover’s algorithm. To test their method, they used a dataset of 10 letters as shown in Figure 7, in order to directly compare the result with them, a variant of the letters dataset was created to match the letter J.

**Figure 8:**
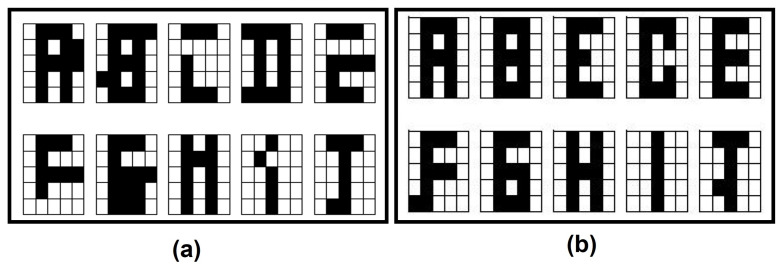
(**a**) Figure designed from the results obtained in [24]. (**b**) The obtained results by the proposed Alpha-Beta HQAM model.

In Figure 8, item (a) shows the obtained result in [24], item (b) shows the obtained results by the proposed Alpha-Beta HQAM model.

By comparison, for the proposed Alpha-Beta HQAM model, there was a completely correct retrieval for six of the ten letters, while they did not retrieve any correctly, and with respect to the others it is noticeable at a glance that there are fewer incorrect pixels, so it can be said that, in general, the proposed model in the retrieval is better than the one reported in [24] It should be noted that the retrieval phase proposed in the new model is less complex to implement, since only the Quantum Hamming Distance algorithm is used, compared to the others which need three algorithms, increasing the number of quantum gates and more qubits needed

### 3.4. Noisy Input Data

To further experiment with the proposed memory, different types of noise were applied at 8% (Additive, Subtractive and Mixed) to the original datasets shown in Figure 4. In order to easily identify the type of noise applied, pixels with Additive noise are shown in gray and pixels with Subtractive noise in blue.

**Figure 9:**
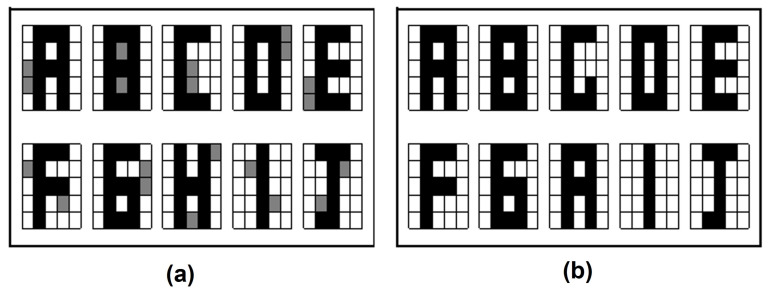
Letters dataset: (**a**) Patterns with additive noise. (**b**) Results of the retrieval.

For Additive Noise, in the letters dataset, Figure 9 in (a) shows the set to be recovered with noisy, and (b) shows the result obtained by the proposed Alpha-Beta HQAM model. The letters that could not be recovered were {C,H}, but for the others the recovery was completely correct. It is possible that it was influenced by the fact that the patterns to be recovered had no loss of information in the qubits that make up the letters.

**Figure 10:**
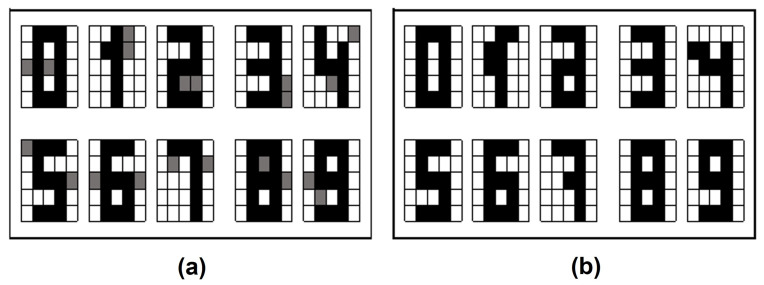
Digit numbers dataset: (**a**) Patterns with aditive noise. (**b**) Results of the retrieval.

In the digit numbers dataset, Figure 10 in (a) shows the set to be recovered with noisy, and in the same Figure 10 but in (b), the result obtained by the proposed Alpha-Beta HQAM model is shown. Only {0,3,5,6,8,9} could be recovered and {2,4,7} only varied by one and two pixels respectively, but {1} only had a recovery of half of the original pattern.

**Figure 11:**
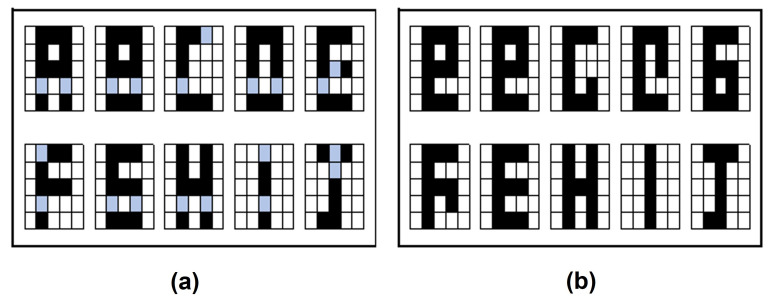
Letters dataset: (**a**) Patterns with subtractive noise. (**b**) Results of the retrieval.

For the Subtractive noise, in the letters dataset, the result was not as expected since only three letters $\left\{H,I,J\right\}$ were completely recovered, although for the others one $\left\{C,D,E,F,G\right\}$ or two $\left\{A\right\}$ pixels varied. The results are shown in Figure 11, in (a) are the patterns with subtractive noise and in (b) are the results of the retrieval.

**Figure 12:**
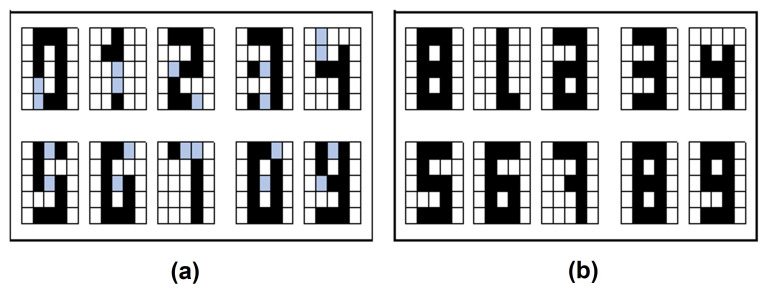
Digit numbers dataset: (**a**) Patterns with subtractive noise. (**b**) Results of the retrieval.

In the digit numbers dataset, the results were favorable because five were complete retrieval {3,5,6,8,9}. However, for {0,2,4,7} it only varied in one pixel and for {1} it failed in two pixels. The results are shown in Figure 12, in (a) are the patterns with subtractive noise and in (b) are the results of the retrieval.

**Figure 13:**
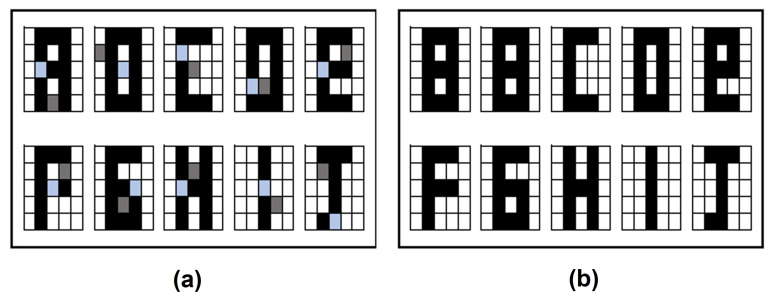
Letters dataset: (**a**) Patterns with mixed noise. (**b**) Results of the retrieval.

Lastly, for the mixed noise, in the letters dataset, a completely correct retrieval was only obtained for $\left\{B,C,D,F,G,H,I,J\right\}$, in the case of $\left\{A\right\}$ there was only a variation in one pixel. The results are shown in Figure 13, in (a) are the patterns with mixed noise and in (b) are the results of the retrieval.

**Figure 14:**
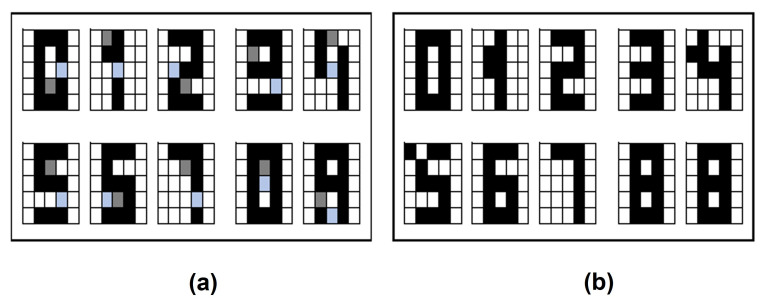
Digit numbers dataset: (**a**) Patterns with mixed noise. (**b**) Results of the retrieval.

In addition, for the digit numbers dataset, $\left\{0,2,3,6,7,8\right\}$ were retrieved completely correctly, but $\left\{1,4,5,9\right\}$ only failed in one pixel, so it is very close to a perfect retrieval. The results are shown in Figure 14, in (a) are the patterns with mixed noise and in (b) are the results of the retrieval.

Additionally, Table 6 and Table 7 show a summary of the results obtained in all the experiments described in the previous sections, these performances are calculated as the percentage of correctly recovered pixels.

## 4. Conclusions

In this work, a hybrid quantum associative memory was proposed and tested on different datasets. The two main attributes of the proposal are the dimensionality reduction of the input patterns using the Alpha-Beta support vector subroutine, which allows the algorithm to run on currently available quantum hardware; and the use of a quantum subroutine to calculate the Hamming distance in the retrieval phase of the memory. The presented results were obtained using IBM’s Qiskit SDK, and show a competitive performance compared to other state-of-the-art works.

It is important to note that the overall quantum complexity of Alpha-Beta HQAM is precisely because the memory has to be reconstructed for each retrieval. This is a widespread problem among quantum algorithms, since the cost of constructing a specific superposition is high. However, it is worth mentioning that Alpha-Beta HQAM does have an advantage over classical memory for retrievals of less than one training. Specifically, for a single retrieval, the advantage is huge: O(n) versus O(mn). This advantage can be used for specific applications in quantum noise correction, for example, if an algorithm is run once, then memory can be used to recover the noise-free version of the output.

An interesting potential extension of this proposal includes the realization of experiments with patterns of higher dimension (more attributes) in order to analyze the retrieval performance of the memory in datasets with this specific feature, and even include efficient initialization protocols to reduce quantum complexity in the training phase.

## Figures and Tables

**Table 1:** Operators *α* and *β*.

*α*: A × A → B			*β*: B × A → A		
*x*	*y*	*α*(*x*,*y*)	*x*	*y*	*β*(*x*,*y*)
0	0	1	0	0	0
0	1	0	0	1	0
1	0	2	1	0	1
1	1	1	1	1	1
			2	0	1
			2	1	1

**Table 2:** Number of features in the letters dataset with the resubstitution error as validation method.

	A	B	C	D	E	F	G	H	I	J
Restricted Fundamental Set	25	25	25	25	25	25	25	25	25	25
Negated Restricted Fundamental Set	15	15	15	15	15	15	15	15	15	15

**Table 3:** Number of features in the digit numbers dataset with the resubstitution error as validation method.

	0	1	2	3	4	5	6	7	8	9
Restricted Fundamental Set	25	25	25	25	25	25	25	25	25	25
Negated Restricted Fundamental Set	15	15	15	15	15	15	15	15	15	15

**Table 4:** Number of features in the letters dataset with the Leave One Out as validation method.

	A	B	C	D	E	F	G	H	I	J
Restricted Fundamental Set	25	25	25	25	25	25	25	24	22	25
Negated Restricted Fundamental Set	15	15	15	15	15	15	15	15	15	15

**Table 5:** Number of features in the digit numbers dataset with the Leave One Out as validation method.

	0	1	2	3	4	5	6	7	8	9
Restricted Fundamental Set	25	22	25	25	24	25	25	24	21	25
Negated Restricted Fundamental Set	15	15	15	15	15	15	15	15	15	15

**Table 6:** Summary table of the accuracy (%) obtained in the experiments of the letters dataset.

	A	B	C	D	E	F	G	H	I	J
Resubstitution Error	100	100	100	100	100	100	100	100	100	100
Leave One Out	100	100	96	100	100	96	100	100	100	96
Additive Noise	100	100	96	100	100	100	100	96	100	100
Substractive Noise	92	96	96	96	96	96	96	100	100	100
Mixed Noise	96	100	100	100	96	100	100	100	100	100

**Table 7:** Summary table of the accuracy (%) obtained in the experiments of the digit numbers dataset.

	0	1	2	3	4	5	6	7	8	9
Resubstitution Error	100	100	100	100	100	100	100	100	100	100
Leave One Out	100	12	88	100	92	100	100	88	100	100
Additive Noise	100	92	96	100	92	100	100	96	100	100
Substractive Noise	96	92	96	100	96	100	100	96	100	100
Mixed Noise	100	96	100	100	96	92	100	100	100	96

**Figure A1:**
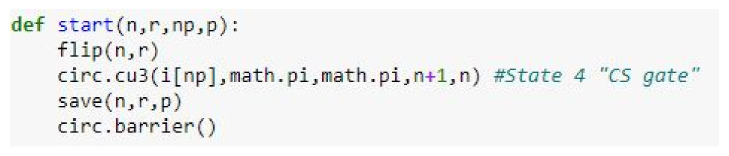
Start function.

**Figure A2:**
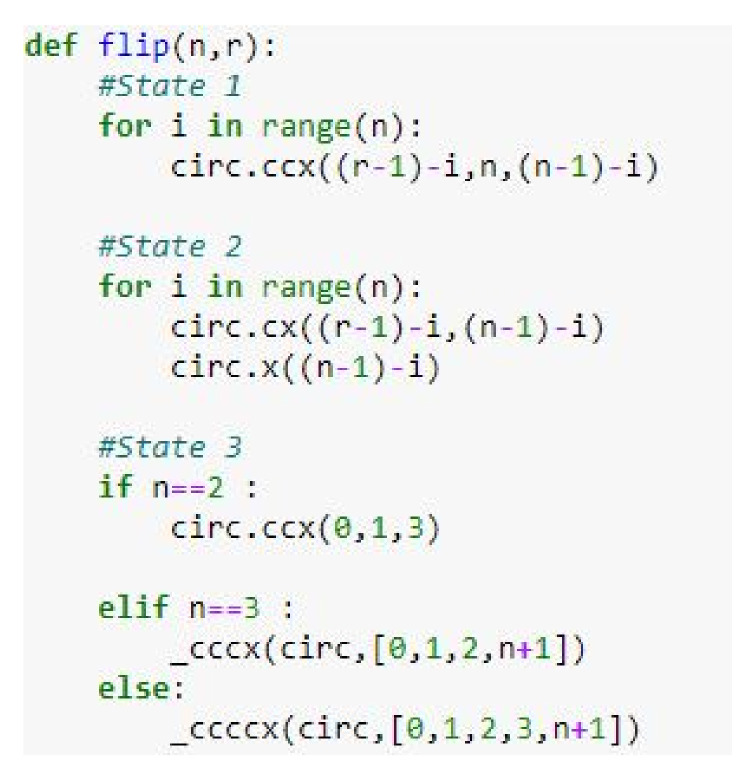
Flip function.

**Figure A3:**
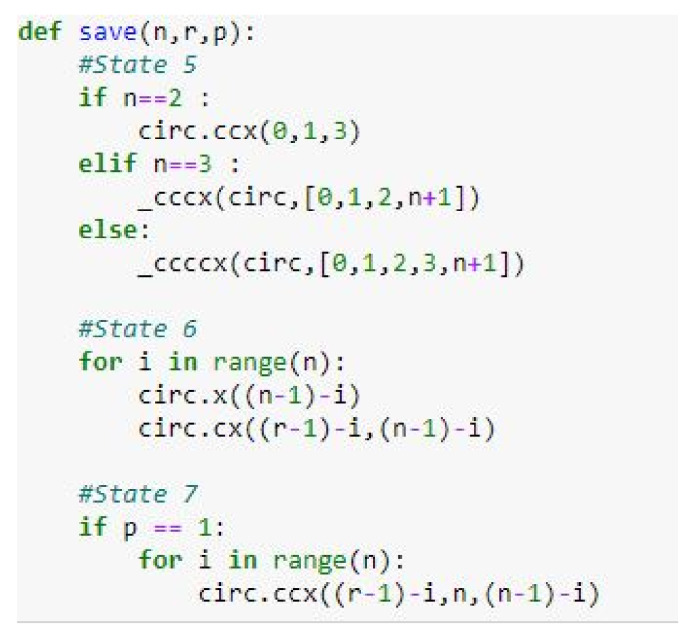
Save function.

**Figure A4:**
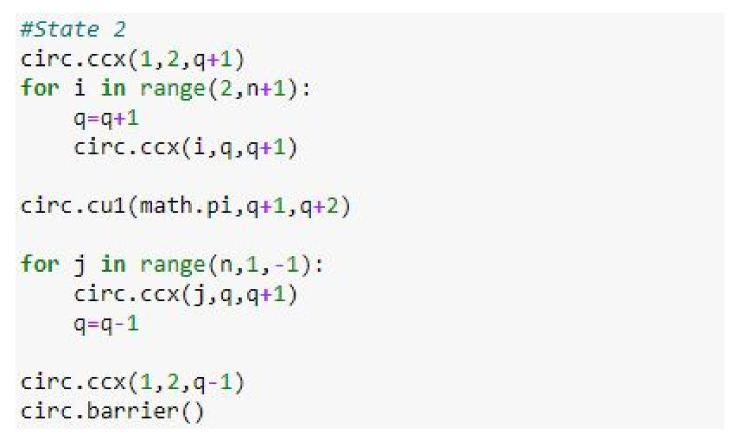
State 2.

**Figure A5:**
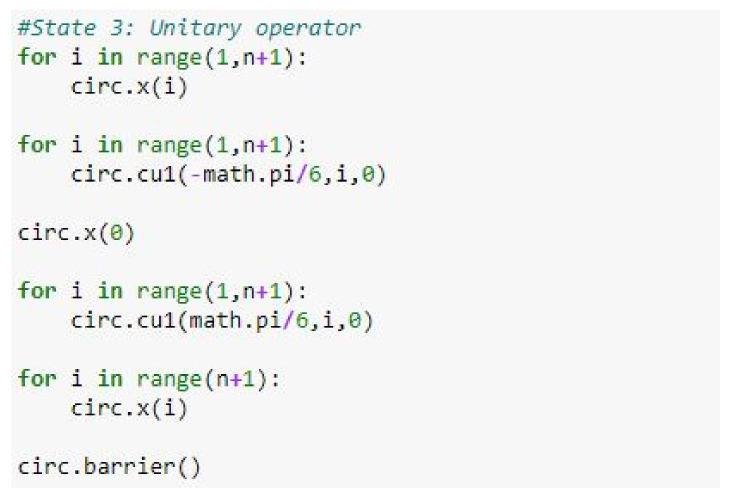
State 3.

**Figure A6:**
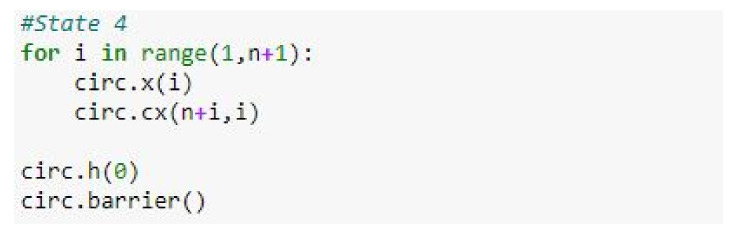
State 4.

## Data Availability

Not applicable.

## Abbreviations

The following abbreviations are used in this manuscript:

QAM	Quantum Associative Memory
SDK	Software Development Kit
RE	Resubstitution Error
SIP	Secretaria de Investigación y Posgrado
IPN	Instituto Politécnico Nacional

## Appendix A. Qiskit Python Code for Experiment Execution

### Appendix A.1. Training Phase

For the training phase, it was divided into three functions based on the original idea of Ventura et al. [7] where he partitioned the algorithm: flip, save and start, which is the main body of the code and includes the two previous ones, as well as the configuration of the CS gate. The functions generated in Qiskit SDK are shown below (Figure A1, Figure A2 and Figure A3):

Where:

*n* = number of qubits

*r* = number of qubits needed for calculations

*np* = parameter to know which number is the pattern being initialized $\left(2*n+2\right)$

*p* = parameter to know if it is the last pattern to be stored, since state 7 is not executed in the last one

### Appendix A.2. Retrieval Phase

For the retrieval phase, it was again divided based on the order of the states for better understanding. As explained in Section 2.6, the initial state consists of three registers, the first contains the input pattern; the second contains the memory **M**, and the third contains the ancilla qubit with initial set to |0〉. The code for the Hamming Distance calculation is shown below (Figure A4, Figure A5 and Figure A6):
